# Crystal structures of two 4*H*-chromene derivatives: 2-amino-3-cyano-4-(3,4-di­chloro­phen­yl)-7-hy­droxy-4*H*-benzo[1,2-*b*]pyran 1,4-dioxane monosolvate and 2-amino-3-cyano-4-(2,6-di­chloro­phen­yl)-7-hy­droxy-4*H*-benzo[1,2-*b*]pyran

**DOI:** 10.1107/S2056989019013197

**Published:** 2019-09-27

**Authors:** S. Silambarasan, A. Jamal Abdul Nasser, T. Mohandas

**Affiliations:** a Department of Chemistry, Jamal Mohamed College, Tiruchirappalli 620 020, India; b Department of Physics, K. Ramakrishnan College of Engineering, Samayapuram, Tiruchirappalli 621 115, India

**Keywords:** crystal structure, 4*H*-chromene, π-conjugation, hydrogen bond, solvate

## Abstract

In the title compounds, C_16_H_9_Cl_2_N_2_O_2_·C_4_H_8_O_2_ and C_16_H_9_Cl_2_N_2_O_2_, the bicyclic 4*H*-chromene cores are nearly planar with maximum deviations of 0.081 (2) and 0.087 (2) Å. In both structures, the chromene derivative mol­ecules are linked into centrosymmetric dimers by pairs of N—H⋯O hydrogen bonds, forming *R^2^_2_*(16) motifs. These dimers are further linked in the 3,4-di­chloro­phenyl derivative by N—H⋯N hydrogen bonds into double layers parallel to (100) and in the 2,6-di­chloro­phenyl derivative by O—H⋯N hydrogen bonds into ribbons along the [10] direction. In the 3,4-di­chloro­phenyl derivative, the 1,4-dioxane solvent mol­ecules are connected to the chromene mol­ecules *via* O—H⋯O hydrogen bonds.

## Abstract 2

In the structures of two title compounds, the 4*H*-chromene derivative mol­ecules are linked by N—H⋯O and N—H⋯N hydrogen bonds, forming double layers or ribbons.

## Chemical context   

Many compounds containing the heterocyclic pyran moiety exhibit diverse pharmacological activities. The pyran ring is a core unit found in benzo­pyrans, chromones, coumarins and flavanoids. Numerous naturally occurring compounds containing a pyran ring show therapeutic activities such as anti­viral (Martínez-Grau & Marco, 1997 ▸), anti­microbial (Khafagy *et al.*, 2002 ▸), mutagenicity (Hiramoto *et al.*, 1997 ▸), sex pheromone (Bianchi & Tava, 1987 ▸), anti­tumor (Mohr *et al.*, 1975 ▸), cancer therapy (Anderson *et al.*, 2005 ▸), central nervous system activity (Eiden & Denk, 1991 ▸), anti­fungal (Schiller *et al.*, 2010 ▸), anti­proliferative (Osman *et al.*, 2011 ▸), anti­diabetic (Bisht *et al.*, 2011 ▸), anti-inflammatory (Wang *et al.*, 1996 ▸; Wang *et al.*, 2005 ▸) and calcium channel antagonist activity (Shahrisa *et al.*, 2011 ▸).

2-Amino-4*H*-benzo[1,2-*b*]pyrans (2-amino-4*H*-chromenes) act as synthetic building blocks for the design of various pyran-containing bio-active mol­ecules (Kale *et al.*, 2013 ▸; Sabry *et al.*, 2011 ▸; Kidwai *et al.*, 2010 ▸); among them are cytotoxic and anti-HIV preparations (Patil *et al.*, 1993 ▸; Emmadi *et al.*, 2012 ▸) and anti­cancer (Wu *et al.*, 2003 ▸; Perrella *et al.*, 1994 ▸), anti­microbial (Mungra *et al.*, 2011 ▸) and anti­coagulant agents (Cingolani *et al.*, 1969 ▸).

Against this background we carried out the crystallographic studies of the title 4*H*-chromenes 2-amino-3-cyano-4-(3,4-di­chloro­phen­yl)-7-hy­droxy-4*H*-benzo[1,2-*b*]pyran 1,4-dioxane mono solvate (I) and 2-amino-3-cyano-4-(2,6-di­chloro­phen­yl)-7-hy­droxy-4*H*-benzo[1,2-*b*]pyran (II).
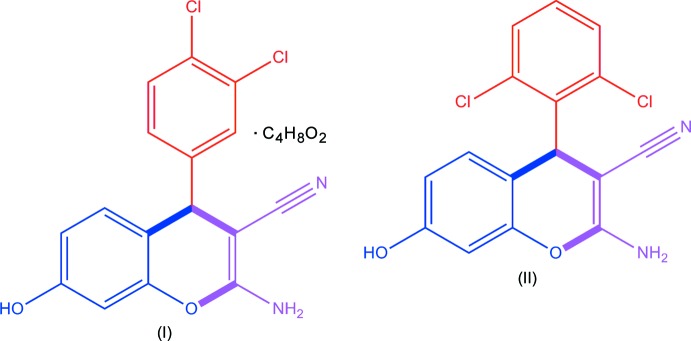


## Structural commentary   

**Figure 1:**
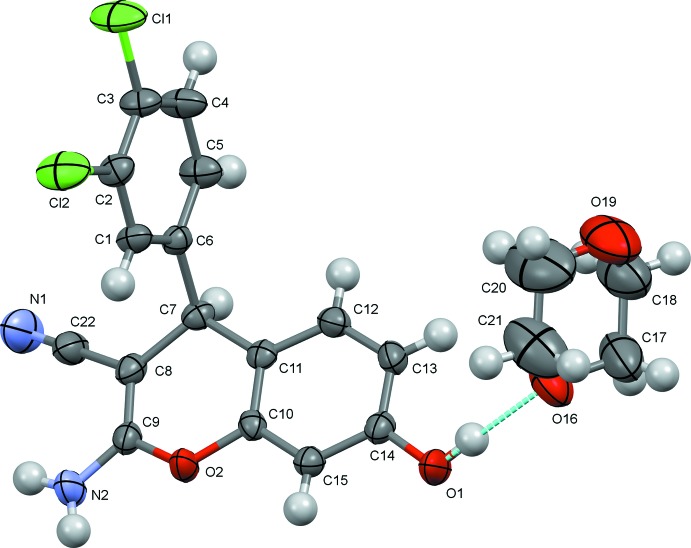
The asymmetric unit of (I), with atom labelling and 50% probability displacement ellipsoids. The hydrogen bond is represented by a dashed line.

The asymmetric units of the title compounds are illustrated in Figs. 1 ▸ and 2 ▸. The mol­ecules of the two 4*H*-chromene deriv­atives differ only in the positions of the chlorine atoms attached to the phenyl ring, and the key bond dimensions in (I) and (II) essentially coincide. The bicyclic chromene cores in the two structures are nearly planar, with the largest deviation from the mean plane being observed for the *sp* ^3^-hybridized C7 atom in both cases [0.081 (2) and 0.087 (2) Å in (I) and (II), respectively]. The inter­atomic distances in the pyran rings indicate a strong π-conjugation of the electron-donating atoms O2 and N2 with the cyano acceptor groups. As a result of this conjugation, the C8=C9 bonds [1.349 (3) Å in (I) and 1.354 (2) Å in (II)] are longer than the typical double bonds (Allen *et al.*, 1987 ▸), whereas the C9—N2 bonds are shortened [1.335 (3) and 1.342 (2) Å for (I) and (II), respectively], thus the amino groups in the studied structures were assumed to be planar and treated with the AFIX 43 instruction. Besides this, the O—C distances in the pyran rings are asymmetric [1.393 (2) and 1.393 (2) Å for O2—C10 *vs*. 1.357 (3) and 1.353 (2) Å for O2—C9 in (I) and (II), respectively]. The observed planarity of the bicyclic chromene units is also a consequence of π-conjugation. The dihedral angle between the mean planes of the 4*H*-chromene ring system and the phenyl ring attached to C7 is 80.82 (9)° in (I) and 85.36 (8)° in (II). In (II), the *o*-chlorine atom Cl1 forms short intra­molecular contacts with atoms C8 and C9 of the pyran ring of 3.111 (2) and 3.193 (2) Å, respectively.

## Supra­molecular features   

**Figure 3:**
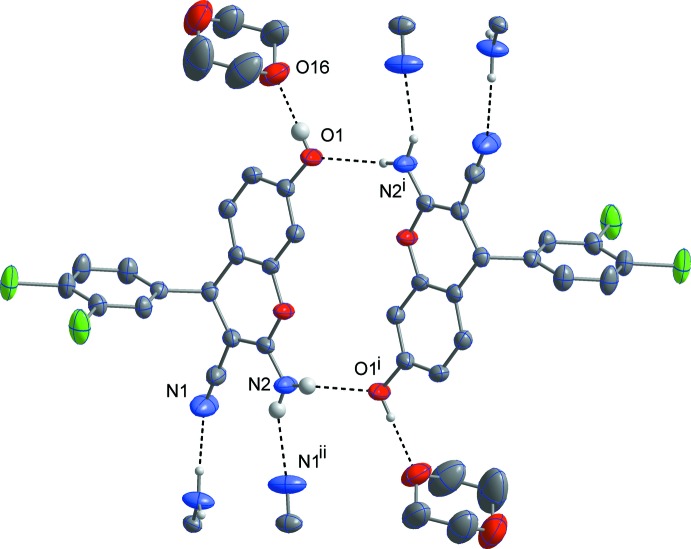
A view of the hydrogen-bonding inter­actions in (I), showing the formation of centrosymmetric dimers. The C-bound hydrogen atoms are omitted for clarity. [Symmetry codes: (i) −*x*, 2 − *y*, 1 − *z*; (ii) −*x*, 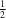 + *y*, 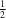 − *z*.]

**Figure 5:**
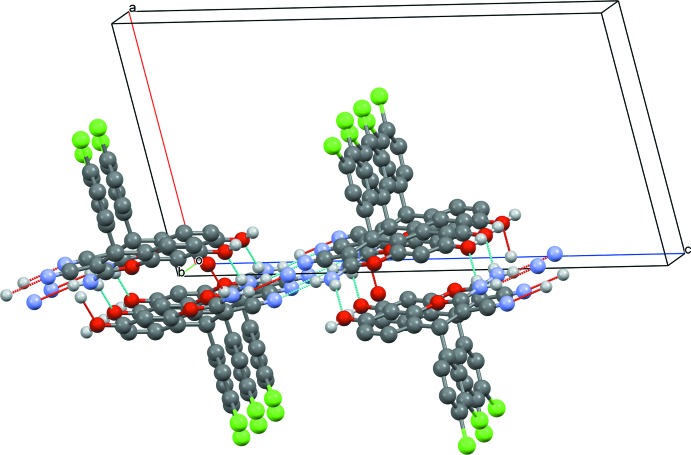
Double layers of hydrogen-bonded mol­ecules in (I). The 1,4-dioxane mol­ecules are omitted for clarity.

As shown in Figs. 3 ▸ and 4 ▸, in both of the title structures the 4*H*-chromene derivative mol­ecules are linked by pairs of N—H⋯O hydrogen bonds (Tables 1 ▸ and 2 ▸) into centrosymmetric dimers, thus forming *R^2^_2_*(16) motifs. In (I), these dimers are further connected by N—H⋯N hydrogen bonds into double layers parallel to the (100) plane (Fig. 5 ▸). In (II), the dimers are linked by O—H⋯N hydrogen bonds into ribbons along the [1
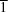
0] direction. In (I), the 1,4-dioxane solvent mol­ecules are linked to the chromene mol­ecules *via* O—H⋯O hydrogen bonds. In this compound, the C—H⋯Cl contacts (Table 2 ▸) also contribute to the stability of crystal structure.

## Database survey   

A search in the Cambridge Structural Database (CSD version 5.40, last update August 2019; Groom *et al.*, 2016 ▸), revealed 107 structures of 4*H*-chromene derivatives, among them 25 containing the 2-amino-3-cyano-4*H*-chromene moiety. Of these, two structures, *viz*. 2-amino-7-hy­droxy-4-(4-hy­droxy­phen­yl)-4*H*-chromene-3-carbo­nitrile (HUPCEC; Horton *et al.*, 2015 ▸) and 2-amino-4-(4-bromo­phen­yl)-7-hy­droxy-4*H*-chromene-3-carbo­nitrile (UFEKOI; Bi *et al.*, 2017 ▸) are closely related to compounds (I) and (II). In the structures of both HUPCEC and UFEKOI, the mol­ecules adopt the same conformation as in (I) and (II) and also form centrosymmetric dimers by pairs of N—H⋯O hydrogen bonds as in the title structures.

## Synthesis and crystallization   

Both studied compounds were prepared by the same procedure. Mixtures of 3,4-chloro­benzaldehyde (8.75 g, 0.05 mol) [for (I)] or 2,4-di­chloro­benzaldehyde (8.75 g, 0.05 mol) [for (II)], malono­nitrile (3.3 ml, 0.05 mol) and resorcinol (5.5 g, 0.05 mol) in 150 ml of water were refluxed for about 10-20 minutes in 250 ml round-bottom flasks. The progress of the reaction was monitored by thin layer chromatography using silica gel-G plates. After the product had formed, the reaction mixtures were kept in the refrigerator overnight. The solid mass that settled was filtered using a suction pump, washed well with a mixture of methanol and water and dried in air. The crude products were recrystallized from methanol giving white powders. Single crystals were grown by slow evaporation of solutions in 1,4-dioxane (I) or aceto­nitrile (II). The melting points are 518-523 K for (I) and 513-515 K for (II).

## Refinement   

Crystal data, diffraction data and structure refinement details for (I) and (II) are summarized in Table 3 ▸. All hydrogen atoms bound to C and N were located from the difference-Fourier maps and refined isotropically using a riding model, with *U*_iso_(H) = 1.2*U*_eq_(C,N) and C—H = 0.98 Å for methine, 0.97 Å for methyl­ene and 0.93 Å for aromatic C atoms, and N—H = 0.86 Å. In (I), the hy­droxy H atom was constrained with AFIX 147, but its *U*_iso_ value was allowed to refine freely. In (II), the OH hydrogen atom was freely refined.

## Supplementary Material

Crystal structure: contains datablock(s) I, II, global. DOI: 10.1107/S2056989019013197/yk2126sup1.cif

Structure factors: contains datablock(s) I. DOI: 10.1107/S2056989019013197/yk2126Isup2.hkl

Structure factors: contains datablock(s) II. DOI: 10.1107/S2056989019013197/yk2126IIsup3.hkl

Supporting information file. DOI: 10.1107/S2056989019013197/yk2126Isup4.cml

Supporting information file. DOI: 10.1107/S2056989019013197/yk2126IIsup5.cml

CCDC references: 1865757, 1865762

Additional supporting information:  crystallographic information; 3D view; checkCIF report

## Figures and Tables

**Figure 2:**
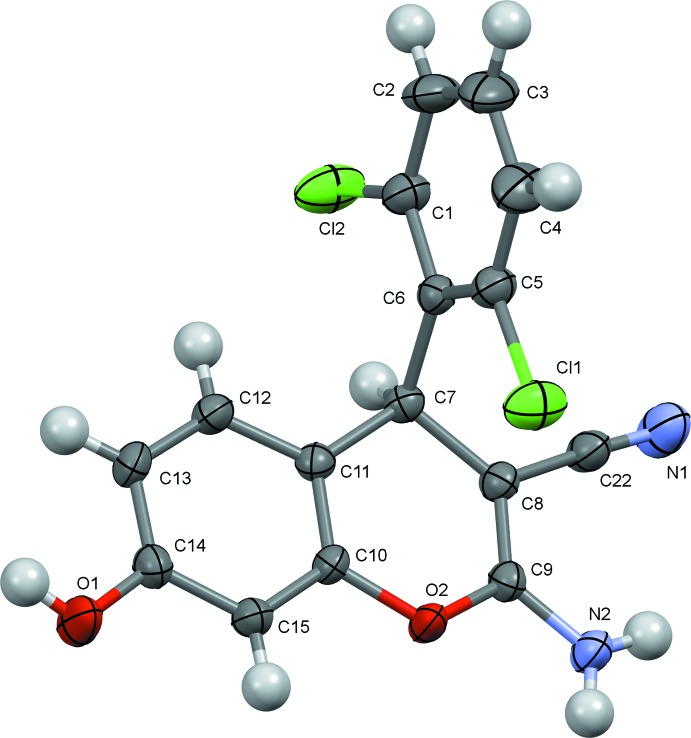
The mol­ecular structure of (II), with atom labelling and 50% probability displacement ellipsoids.

**Figure 4:**
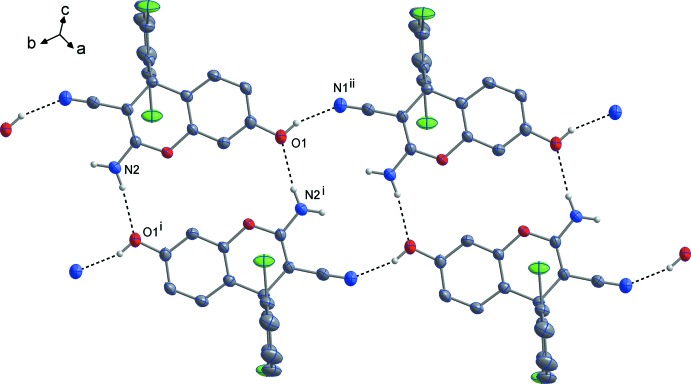
A view of the hydrogen-bonding inter­actions in (II), showing the formation of centrosymmetric dimers and ribbons. The C-bound hydrogen atoms are omitted for clarity. [Symmetry codes: (i) 2 − *x*, 1 − *y*, −*z*; (ii) 1 + *x*, *y* − 1, *z*.]

**Table 1:** Hydrogen-bond geometry (Å, °) for (I)

*D*—H⋯*A*	*D*—H	H⋯*A*	*D*⋯*A*	*D*—H⋯*A*
O1—H*O*1⋯O16	0.82	1.85	2.658 (3)	167
N2—H*NB*⋯O1^i^	0.86	2.19	2.978 (3)	153
N2—H*NA*⋯N1^ii^	0.86	2.22	2.989 (3)	149
C4—H4⋯Cl2^iii^	0.93	2.87	3.692 (3)	148

Symmetry codes: (i) 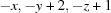; (ii) 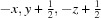; (iii) 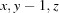.

**Table 2:** Hydrogen-bond geometry (Å, °) for (II)

*D*—H⋯*A*	*D*—H	H⋯*A*	*D*⋯*A*	*D*—H⋯*A*
C7—H7⋯Cl2	0.98	2.50	3.078 (3)	117
N2—H*NB*⋯O1^i^	0.86	2.19	3.048 (2)	173
O1—H*O*1⋯N1^ii^	0.85 (3)	1.95 (3)	2.762 (2)	160 (3)

Symmetry codes: (i) 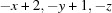; (ii) 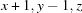.

**Table 3:** Experimental details

	(I)	(II)
Crystal data		
Chemical formula	C_16_H_9_Cl_2_N_2_O_2_·C_4_H_8_O_2_	C_16_H_9_Cl_2_N_2_O_2_
*M*_r_	420.25	332.15
Crystal system, space group	Monoclinic, *P*2_1_/*c*	Triclinic, *P* 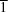
Temperature (K)	294	294
*a*, *b*, *c* (Å)	12.753 (9), 6.665 (4), 24.050 (14)	6.271 (3), 8.697 (5), 13.794 (7)
α, β, γ (°)	90, 102.95 (3), 90	107.06 (2), 94.269 (17), 95.00 (3)
*V* (Å^3^)	1992 (2)	712.5 (7)
*Z*	4	2
Radiation type	Mo *K*α	Mo *K*α
μ (mm^−1^)	0.36	0.46
Crystal size (mm)	0.15 × 0.15 × 0.10	0.15 × 0.15 × 0.10

Data collection		
Diffractometer	Bruker Kappa APEX3 CMOS	Bruker Kappa APEX3 CMOS
Absorption correction	Multi-scan (*SADABS*; Bruker, 2018 ▸)	Multi-scan (*SADABS*; Bruker, 2018 ▸)
*T*_min_, *T*_max_	0.704, 0.745	0.704, 0.746
No. of measured, independent and observed [*I* > 2σ(*I*)] reflections	34682, 3492, 2885	22289, 2499, 2239
*R*_int_	0.033	0.023
(sin θ/λ)_max_ (Å^−1^)	0.595	0.595

Refinement		
*R*[*F* ^2^ > 2σ(*F* ^2^)], *wR*(*F* ^2^), *S*	0.044, 0.121, 1.11	0.033, 0.081, 1.11
No. of reflections	3492	2499
No. of parameters	256	204
H-atom treatment	H atoms treated by a mixture of independent and constrained refinement	H atoms treated by a mixture of independent and constrained refinement
Δρ_max_, Δρ_min_ (e Å^−3^)	0.26, −0.24	0.23, −0.25

Computer programs: *APEX3*, *SAINT* and *XPREP* (Bruker, 2018 ▸), *SHELXT2014/5* (Sheldrick, 2015*a* ▸), *SHELXL2014/6* (Sheldrick, 2015*b* ▸), *DIAMOND* (Brandenburg, 2007 ▸), *Mercury* (Macrae *et al.*, 2008 ▸) and *PLATON* (Spek, 2009 ▸).

## supplementary crystallographic information

### 2-Amino-3-cyano-4-(3,4-dichlorophenyl)-7-hydroxy-4H-benzo[1,2-b]pyran 1,4-dioxane monosolvate (I) Crystal data

**Table:**

C_16_H_9_Cl_2_N_2_O_2_·C_4_H_8_O_2_	*D*_x_ = 1.401 Mg m^−^^3^
*M**_r_* = 420.25	Melting point: 520 K
Monoclinic, *P*2_1_/*c*	Mo *K*α radiation, λ = 0.71073 Å
*a* = 12.753 (9) Å	Cell parameters from 9965 reflections
*b* = 6.665 (4) Å	θ = 3.2–26.7°
*c* = 24.050 (14) Å	µ = 0.36 mm^−^^1^
β = 102.95 (3)°	*T* = 294 K
*V* = 1992 (2) Å^3^	Block, colourless
*Z* = 4	0.15 × 0.15 × 0.10 mm
*F*(000) = 868	

### 2-Amino-3-cyano-4-(3,4-dichlorophenyl)-7-hydroxy-4H-benzo[1,2-b]pyran 1,4-dioxane monosolvate (I) Data collection

**Table:**

Bruker Kappa APEX3 CMOS diffractometer	3492 independent reflections
Radiation source: fine-focus sealed tube	2885 reflections with *I* > 2σ(*I*)
Graphite monochromator	*R*_int_ = 0.033
ω and φ scan	θ_max_ = 25.0°, θ_min_ = 3.2°
Absorption correction: multi-scan (SADABS; Bruker, 2018)	*h* = −15→15
*T*_min_ = 0.704, *T*_max_ = 0.745	*k* = −7→7
34682 measured reflections	*l* = −28→26

### 2-Amino-3-cyano-4-(3,4-dichlorophenyl)-7-hydroxy-4H-benzo[1,2-b]pyran 1,4-dioxane monosolvate (I) Refinement

**Table:**

Refinement on *F*^2^	Secondary atom site location: difference Fourier map
Least-squares matrix: full	Hydrogen site location: difference Fourier map
*R*[*F*^2^ > 2σ(*F*^2^)] = 0.044	H atoms treated by a mixture of independent and constrained refinement
*wR*(*F*^2^) = 0.121	*w* = 1/[σ^2^(*F*_o_^2^) + (0.0541*P*)^2^ + 1.1852*P*] where *P* = (*F*_o_^2^ + 2*F*_c_^2^)/3
*S* = 1.11	(Δ/σ)_max_ < 0.001
3492 reflections	Δρ_max_ = 0.26 e Å^−^^3^
256 parameters	Δρ_min_ = −0.24 e Å^−^^3^
0 restraints	Extinction correction: SHELXL, Fc^*^=kFc[1+0.001xFc^2^λ^3^/sin(2θ)]^-1/4^
Primary atom site location: structure-invariant direct methods	Extinction coefficient: 0.0174 (18)

### 2-Amino-3-cyano-4-(3,4-dichlorophenyl)-7-hydroxy-4H-benzo[1,2-b]pyran 1,4-dioxane monosolvate (I) Special details

**Table:**

Geometry. All esds (except the esd in the dihedral angle between two l.s. planes) are estimated using the full covariance matrix. The cell esds are taken into account individually in the estimation of esds in distances, angles and torsion angles; correlations between esds in cell parameters are only used when they are defined by crystal symmetry. An approximate (isotropic) treatment of cell esds is used for estimating esds involving l.s. planes.
Refinement. Refinement of F^2^ against ALL reflections. The weighted R-factor wR and goodness of fit S are based on F^2^, conventional R-factors R are based on F, with F set to zero for negative F^2^. The threshold expression of F^2^ > 2sigma(F^2^) is used only for calculating R-factors(gt) etc. and is not relevant to the choice of reflections for refinement. R-factors based on F^2^ are statistically about twice as large as those based on F, and R- factors based on ALL data will be even larger.

### 2-Amino-3-cyano-4-(3,4-dichlorophenyl)-7-hydroxy-4H-benzo[1,2-b]pyran 1,4-dioxane monosolvate (I) Fractional atomic coordinates and isotropic or equivalent isotropic displacement parameters (Å^2^)

**Table:**

	*x*	*y*	*z*	*U*_iso_*/*U*_eq_	
Cl1	0.60648 (5)	0.18411 (11)	0.42962 (4)	0.0702 (3)	
Cl2	0.52933 (5)	0.63066 (11)	0.40894 (4)	0.0679 (3)	
O1	0.12386 (15)	0.8018 (3)	0.63654 (6)	0.0519 (5)	
HO1	0.1545	0.7386	0.6648	0.074 (10)*	
O2	0.07140 (12)	0.8197 (2)	0.43851 (6)	0.0379 (4)	
N2	0.01565 (18)	0.8571 (3)	0.34552 (8)	0.0530 (6)	
HNA	0.0051	0.8207	0.3104	0.064*	
HNB	−0.0032	0.9750	0.3540	0.064*	
N1	0.0608 (2)	0.3924 (4)	0.28098 (9)	0.0748 (8)	
C3	0.47549 (17)	0.2502 (4)	0.42953 (10)	0.0424 (6)	
C4	0.40418 (19)	0.1065 (4)	0.43879 (12)	0.0512 (6)	
H4	0.4266	−0.0260	0.4452	0.061*	
C5	0.29931 (18)	0.1579 (3)	0.43861 (11)	0.0439 (6)	
H5	0.2516	0.0592	0.4448	0.053*	
C6	0.26401 (16)	0.3532 (3)	0.42946 (8)	0.0303 (5)	
C7	0.14837 (15)	0.4087 (3)	0.43059 (8)	0.0305 (5)	
H7	0.1068	0.2841	0.4279	0.037*	
C11	0.14231 (15)	0.5105 (3)	0.48595 (8)	0.0292 (4)	
C12	0.17291 (16)	0.4127 (3)	0.53832 (9)	0.0358 (5)	
H12	0.1980	0.2815	0.5391	0.043*	
C13	0.16731 (17)	0.5037 (4)	0.58889 (9)	0.0382 (5)	
H13	0.1868	0.4337	0.6231	0.046*	
C14	0.13242 (16)	0.7003 (3)	0.58846 (8)	0.0354 (5)	
C10	0.10667 (15)	0.7052 (3)	0.48756 (8)	0.0291 (4)	
C15	0.10195 (16)	0.8023 (3)	0.53756 (8)	0.0337 (5)	
H15	0.0786	0.9346	0.5369	0.040*	
C9	0.06130 (16)	0.7304 (3)	0.38688 (8)	0.0342 (5)	
C8	0.09544 (16)	0.5416 (3)	0.38111 (8)	0.0347 (5)	
C22	0.07678 (19)	0.4596 (4)	0.32560 (10)	0.0459 (6)	
C1	0.33627 (17)	0.4972 (3)	0.42043 (9)	0.0361 (5)	
H1	0.3140	0.6298	0.4143	0.043*	
C2	0.44144 (17)	0.4463 (3)	0.42042 (9)	0.0380 (5)	
O16	0.24114 (17)	0.6481 (4)	0.73252 (9)	0.0769 (6)	
C17	0.2288 (3)	0.5035 (7)	0.77295 (16)	0.0937 (12)	
H171	0.1565	0.4491	0.7630	0.112*	
H172	0.2389	0.5659	0.8102	0.112*	
C18	0.3066 (3)	0.3413 (7)	0.7754 (2)	0.1008 (13)	
H181	0.2976	0.2450	0.8042	0.121*	
H182	0.2930	0.2727	0.7389	0.121*	
O19	0.4122 (2)	0.4128 (5)	0.78824 (13)	0.1129 (10)	
C20	0.4249 (4)	0.5602 (9)	0.7484 (3)	0.140 (2)	
H201	0.4159	0.4986	0.7111	0.169*	
H202	0.4974	0.6137	0.7590	0.169*	
C21	0.3484 (3)	0.7239 (7)	0.7450 (2)	0.1147 (16)	
H211	0.3622	0.7957	0.7810	0.138*	
H212	0.3573	0.8171	0.7154	0.138*	

### 2-Amino-3-cyano-4-(3,4-dichlorophenyl)-7-hydroxy-4H-benzo[1,2-b]pyran 1,4-dioxane monosolvate (I) Atomic displacement parameters (Å^2^)

**Table:**

	*U*^11^	*U*^22^	*U*^33^	*U*^12^	*U*^13^	*U*^23^
Cl1	0.0349 (3)	0.0613 (5)	0.1189 (7)	0.0130 (3)	0.0268 (4)	0.0064 (4)
Cl2	0.0449 (4)	0.0492 (4)	0.1188 (7)	−0.0051 (3)	0.0379 (4)	0.0059 (4)
O1	0.0682 (12)	0.0615 (11)	0.0250 (8)	0.0233 (9)	0.0080 (8)	−0.0009 (8)
O2	0.0528 (9)	0.0356 (8)	0.0245 (7)	0.0081 (7)	0.0070 (6)	0.0006 (6)
N2	0.0766 (15)	0.0540 (13)	0.0266 (10)	0.0236 (11)	0.0079 (9)	0.0035 (9)
N1	0.100 (2)	0.0820 (18)	0.0351 (13)	0.0287 (16)	−0.0006 (12)	−0.0151 (12)
C3	0.0294 (11)	0.0424 (13)	0.0557 (14)	0.0061 (10)	0.0105 (10)	−0.0031 (11)
C4	0.0408 (13)	0.0324 (12)	0.0819 (18)	0.0077 (10)	0.0171 (12)	0.0016 (12)
C5	0.0362 (12)	0.0333 (12)	0.0634 (15)	−0.0015 (10)	0.0138 (11)	−0.0009 (11)
C6	0.0300 (10)	0.0334 (11)	0.0273 (10)	0.0020 (9)	0.0059 (8)	−0.0045 (9)
C7	0.0258 (10)	0.0325 (11)	0.0328 (11)	−0.0011 (8)	0.0062 (8)	−0.0046 (9)
C11	0.0220 (9)	0.0354 (11)	0.0305 (10)	−0.0020 (8)	0.0062 (8)	−0.0008 (9)
C12	0.0333 (11)	0.0346 (12)	0.0394 (12)	0.0048 (9)	0.0082 (9)	0.0041 (9)
C13	0.0374 (12)	0.0481 (14)	0.0285 (11)	0.0053 (10)	0.0060 (9)	0.0080 (10)
C14	0.0305 (11)	0.0481 (13)	0.0275 (11)	0.0035 (9)	0.0065 (8)	−0.0018 (9)
C10	0.0244 (9)	0.0370 (11)	0.0253 (10)	−0.0007 (8)	0.0042 (8)	0.0035 (9)
C15	0.0323 (11)	0.0377 (12)	0.0306 (11)	0.0046 (9)	0.0061 (8)	−0.0010 (9)
C9	0.0317 (11)	0.0455 (13)	0.0261 (10)	0.0020 (9)	0.0079 (8)	0.0005 (9)
C8	0.0284 (10)	0.0474 (13)	0.0274 (10)	0.0041 (9)	0.0043 (8)	−0.0054 (9)
C22	0.0459 (13)	0.0541 (15)	0.0347 (13)	0.0145 (11)	0.0028 (10)	−0.0041 (11)
C1	0.0344 (11)	0.0306 (11)	0.0450 (12)	0.0042 (9)	0.0126 (9)	−0.0021 (9)
C2	0.0320 (11)	0.0395 (12)	0.0439 (12)	−0.0026 (9)	0.0115 (9)	−0.0032 (10)
O16	0.0632 (13)	0.0935 (16)	0.0644 (13)	0.0044 (12)	−0.0062 (10)	0.0279 (12)
C17	0.067 (2)	0.126 (3)	0.091 (2)	0.018 (2)	0.0234 (18)	0.049 (2)
C18	0.082 (3)	0.096 (3)	0.126 (3)	0.008 (2)	0.027 (2)	0.042 (2)
O19	0.0663 (16)	0.131 (3)	0.137 (2)	0.0268 (17)	0.0141 (15)	0.043 (2)
C20	0.075 (3)	0.139 (4)	0.219 (6)	0.007 (3)	0.056 (3)	0.052 (5)
C21	0.079 (3)	0.108 (3)	0.141 (4)	−0.018 (3)	−0.008 (2)	0.034 (3)

### 2-Amino-3-cyano-4-(3,4-dichlorophenyl)-7-hydroxy-4H-benzo[1,2-b]pyran 1,4-dioxane monosolvate (I) Geometric parameters (Å, º)

**Table:**

Cl1—C3	1.727 (2)	C12—H12	0.9300
Cl2—C2	1.727 (2)	C13—C14	1.383 (3)
O1—C14	1.365 (3)	C13—H13	0.9300
O1—HO1	0.8200	C14—C15	1.378 (3)
O2—C9	1.357 (3)	C10—C15	1.379 (3)
O2—C10	1.393 (2)	C15—H15	0.9300
N2—C9	1.335 (3)	C9—C8	1.349 (3)
N2—HNA	0.8600	C8—C22	1.413 (3)
N2—HNB	0.8600	C1—C2	1.384 (3)
N1—C22	1.138 (3)	C1—H1	0.9300
C3—C4	1.373 (3)	O16—C17	1.403 (4)
C3—C2	1.379 (3)	O16—C21	1.426 (5)
C4—C5	1.380 (3)	C17—C18	1.459 (5)
C4—H4	0.9300	C17—H171	0.9700
C5—C6	1.379 (3)	C17—H172	0.9700
C5—H5	0.9300	C18—O19	1.396 (5)
C6—C1	1.381 (3)	C18—H181	0.9700
C6—C7	1.527 (3)	C18—H182	0.9700
C7—C11	1.512 (3)	O19—C20	1.406 (5)
C7—C8	1.515 (3)	C20—C21	1.453 (7)
C7—H7	0.9800	C20—H201	0.9700
C11—C10	1.378 (3)	C20—H202	0.9700
C11—C12	1.394 (3)	C21—H211	0.9700
C12—C13	1.375 (3)	C21—H212	0.9700
			
C14—O1—HO1	109.5	C10—C15—H15	120.5
C9—O2—C10	118.75 (17)	N2—C9—C8	127.6 (2)
C9—N2—HNA	120.0	N2—C9—O2	109.9 (2)
C9—N2—HNB	120.0	C8—C9—O2	122.48 (19)
HNA—N2—HNB	120.0	C9—C8—C22	117.9 (2)
C4—C3—C2	119.4 (2)	C9—C8—C7	124.17 (18)
C4—C3—Cl1	119.81 (19)	C22—C8—C7	117.8 (2)
C2—C3—Cl1	120.80 (18)	N1—C22—C8	179.3 (3)
C3—C4—C5	120.2 (2)	C6—C1—C2	120.7 (2)
C3—C4—H4	119.9	C6—C1—H1	119.6
C5—C4—H4	119.9	C2—C1—H1	119.6
C6—C5—C4	121.1 (2)	C3—C2—C1	120.2 (2)
C6—C5—H5	119.4	C3—C2—Cl2	120.48 (17)
C4—C5—H5	119.4	C1—C2—Cl2	119.31 (18)
C5—C6—C1	118.41 (19)	C17—O16—C21	110.4 (3)
C5—C6—C7	120.53 (19)	O16—C17—C18	110.9 (3)
C1—C6—C7	121.05 (19)	O16—C17—H171	109.5
C11—C7—C8	109.13 (17)	C18—C17—H171	109.5
C11—C7—C6	111.39 (16)	O16—C17—H172	109.5
C8—C7—C6	112.94 (16)	C18—C17—H172	109.5
C11—C7—H7	107.7	H171—C17—H172	108.1
C8—C7—H7	107.7	O19—C18—C17	111.6 (4)
C6—C7—H7	107.7	O19—C18—H181	109.3
C10—C11—C12	116.24 (18)	C17—C18—H181	109.3
C10—C11—C7	121.97 (18)	O19—C18—H182	109.3
C12—C11—C7	121.79 (19)	C17—C18—H182	109.3
C13—C12—C11	122.2 (2)	H181—C18—H182	108.0
C13—C12—H12	118.9	C18—O19—C20	109.9 (3)
C11—C12—H12	118.9	O19—C20—C21	112.6 (4)
C12—C13—C14	119.5 (2)	O19—C20—H201	109.1
C12—C13—H13	120.3	C21—C20—H201	109.1
C14—C13—H13	120.3	O19—C20—H202	109.1
O1—C14—C15	116.6 (2)	C21—C20—H202	109.1
O1—C14—C13	123.34 (19)	H201—C20—H202	107.8
C15—C14—C13	120.01 (19)	O16—C21—C20	110.2 (4)
C11—C10—C15	123.07 (18)	O16—C21—H211	109.6
C11—C10—O2	122.57 (17)	C20—C21—H211	109.6
C15—C10—O2	114.36 (18)	O16—C21—H212	109.6
C14—C15—C10	119.0 (2)	C20—C21—H212	109.6
C14—C15—H15	120.5	H211—C21—H212	108.1
			
C2—C3—C4—C5	0.4 (4)	C11—C10—C15—C14	−1.1 (3)
Cl1—C3—C4—C5	−179.8 (2)	O2—C10—C15—C14	178.35 (18)
C3—C4—C5—C6	−0.3 (4)	C10—O2—C9—N2	173.17 (18)
C4—C5—C6—C1	0.0 (3)	C10—O2—C9—C8	−7.7 (3)
C4—C5—C6—C7	−178.7 (2)	N2—C9—C8—C22	−3.7 (3)
C5—C6—C7—C11	102.8 (2)	O2—C9—C8—C22	177.2 (2)
C1—C6—C7—C11	−75.9 (2)	N2—C9—C8—C7	178.9 (2)
C5—C6—C7—C8	−134.0 (2)	O2—C9—C8—C7	−0.1 (3)
C1—C6—C7—C8	47.3 (3)	C11—C7—C8—C9	7.6 (3)
C8—C7—C11—C10	−8.1 (2)	C6—C7—C8—C9	−116.9 (2)
C6—C7—C11—C10	117.2 (2)	C11—C7—C8—C22	−169.73 (19)
C8—C7—C11—C12	172.12 (18)	C6—C7—C8—C22	65.8 (2)
C6—C7—C11—C12	−62.5 (2)	C5—C6—C1—C2	0.2 (3)
C10—C11—C12—C13	0.5 (3)	C7—C6—C1—C2	178.92 (19)
C7—C11—C12—C13	−179.75 (19)	C4—C3—C2—C1	−0.2 (4)
C11—C12—C13—C14	−1.4 (3)	Cl1—C3—C2—C1	179.98 (18)
C12—C13—C14—O1	179.6 (2)	C4—C3—C2—Cl2	179.7 (2)
C12—C13—C14—C15	1.1 (3)	Cl1—C3—C2—Cl2	−0.1 (3)
C12—C11—C10—C15	0.8 (3)	C6—C1—C2—C3	−0.1 (3)
C7—C11—C10—C15	−178.98 (18)	C6—C1—C2—Cl2	179.97 (16)
C12—C11—C10—O2	−178.63 (18)	C21—O16—C17—C18	−56.6 (5)
C7—C11—C10—O2	1.6 (3)	O16—C17—C18—O19	57.7 (5)
C9—O2—C10—C11	6.9 (3)	C17—C18—O19—C20	−56.0 (5)
C9—O2—C10—C15	−172.56 (18)	C18—O19—C20—C21	55.8 (6)
O1—C14—C15—C10	−178.52 (19)	C17—O16—C21—C20	55.5 (5)
C13—C14—C15—C10	0.1 (3)	O19—C20—C21—O16	−55.7 (6)

### 2-Amino-3-cyano-4-(3,4-dichlorophenyl)-7-hydroxy-4H-benzo[1,2-b]pyran 1,4-dioxane monosolvate (I) Hydrogen-bond geometry (Å, º)

**Table:**

*D*—H···*A*	*D*—H	H···*A*	*D*···*A*	*D*—H···*A*
O1—H*O*1···O16	0.82	1.85	2.658 (3)	167
N2—H*NB*···O1^i^	0.86	2.19	2.978 (3)	153
N2—H*NA*···N1^ii^	0.86	2.22	2.989 (3)	149
C4—H4···Cl2^iii^	0.93	2.87	3.692 (3)	148

Symmetry codes: (i) −*x*, −*y*+2, −*z*+1; (ii) −*x*, *y*+1/2, −*z*+1/2; (iii) *x*, *y*−1, *z*.

### 2-Amino-3-cyano-4-(2,6-dichlorophenyl)-7-hydroxy-4H-\ benzo[1,2-b]pyran (II) Crystal data

**Table:**

C_16_H_9_Cl_2_N_2_O_2_	*F*(000) = 338
*M**_r_* = 332.15	*D*_x_ = 1.548 Mg m^−^^3^
Triclinic, *P*1	Melting point: 514 K
*a* = 6.271 (3) Å	Mo *K*α radiation, λ = 0.71073 Å
*b* = 8.697 (5) Å	Cell parameters from 8750 reflections
*c* = 13.794 (7) Å	θ = 3.1–30.4°
α = 107.06 (2)°	µ = 0.46 mm^−^^1^
β = 94.269 (17)°	*T* = 294 K
γ = 95.00 (3)°	Block, colourless
*V* = 712.5 (7) Å^3^	0.15 × 0.15 × 0.10 mm
*Z* = 2	

### 2-Amino-3-cyano-4-(2,6-dichlorophenyl)-7-hydroxy-4H-\ benzo[1,2-b]pyran (II) Data collection

**Table:**

Bruker Kappa APEX3 CMOS diffractometer	2499 independent reflections
Radiation source: fine-focus sealed tube	2239 reflections with *I* > 2σ(*I*)
Graphite monochromator	*R*_int_ = 0.023
ω and φ scan	θ_max_ = 25.0°, θ_min_ = 3.9°
Absorption correction: multi-scan (SADABS; Bruker, 2018)	*h* = −7→7
*T*_min_ = 0.704, *T*_max_ = 0.746	*k* = −10→10
22289 measured reflections	*l* = −16→16

### 2-Amino-3-cyano-4-(2,6-dichlorophenyl)-7-hydroxy-4H-\ benzo[1,2-b]pyran (II) Refinement

**Table:**

Refinement on *F*^2^	Secondary atom site location: difference Fourier map
Least-squares matrix: full	Hydrogen site location: mixed
*R*[*F*^2^ > 2σ(*F*^2^)] = 0.033	H atoms treated by a mixture of independent and constrained refinement
*wR*(*F*^2^) = 0.081	*w* = 1/[σ^2^(*F*_o_^2^) + (0.0263*P*)^2^ + 0.4717*P*] where *P* = (*F*_o_^2^ + 2*F*_c_^2^)/3
*S* = 1.11	(Δ/σ)_max_ < 0.001
2499 reflections	Δρ_max_ = 0.23 e Å^−^^3^
204 parameters	Δρ_min_ = −0.25 e Å^−^^3^
0 restraints	Extinction correction: SHELXL, Fc^*^=kFc[1+0.001xFc^2^λ^3^/sin(2θ)]^-1/4^
Primary atom site location: structure-invariant direct methods	Extinction coefficient: 0.080 (10)

### 2-Amino-3-cyano-4-(2,6-dichlorophenyl)-7-hydroxy-4H-\ benzo[1,2-b]pyran (II) Special details

**Table:**

Geometry. All esds (except the esd in the dihedral angle between two l.s. planes) are estimated using the full covariance matrix. The cell esds are taken into account individually in the estimation of esds in distances, angles and torsion angles; correlations between esds in cell parameters are only used when they are defined by crystal symmetry. An approximate (isotropic) treatment of cell esds is used for estimating esds involving l.s. planes.
Refinement. Refinement of F^2^ against ALL reflections. The weighted R-factor wR and goodness of fit S are based on F^2^, conventional R-factors R are based on F, with F set to zero for negative F^2^. The threshold expression of F^2^ > 2sigma(F^2^) is used only for calculating R-factors(gt) etc. and is not relevant to the choice of reflections for refinement. R-factors based on F^2^ are statistically about twice as large as those based on F, and R- factors based on ALL data will be even larger.

### 2-Amino-3-cyano-4-(2,6-dichlorophenyl)-7-hydroxy-4H-\ benzo[1,2-b]pyran (II) Fractional atomic coordinates and isotropic or equivalent isotropic displacement parameters (Å^2^)

**Table:**

	*x*	*y*	*z*	*U*_iso_*/*U*_eq_	
Cl1	0.84692 (8)	0.84534 (6)	0.32241 (4)	0.04703 (19)	
Cl2	0.10752 (8)	0.50064 (7)	0.36100 (4)	0.0536 (2)	
O1	1.0482 (2)	0.15972 (18)	0.09764 (12)	0.0484 (4)	
HO1	1.058 (5)	0.092 (4)	0.130 (2)	0.084 (10)*	
O2	0.7394 (2)	0.62001 (14)	0.07971 (9)	0.0331 (3)	
N1	0.1786 (3)	0.9314 (2)	0.18547 (15)	0.0538 (5)	
N2	0.6119 (3)	0.83118 (18)	0.04811 (12)	0.0372 (4)	
HNB	0.7128	0.8266	0.0087	0.045*	
HNA	0.5262	0.9046	0.0540	0.045*	
C14	0.8930 (3)	0.2578 (2)	0.12922 (13)	0.0314 (4)	
C13	0.7476 (3)	0.2339 (2)	0.19616 (13)	0.0327 (4)	
H13	0.7498	0.1452	0.2207	0.039*	
C12	0.6001 (3)	0.3428 (2)	0.22588 (13)	0.0303 (4)	
H12	0.5033	0.3258	0.2706	0.036*	
C11	0.5912 (3)	0.47743 (19)	0.19116 (12)	0.0252 (4)	
C7	0.4337 (3)	0.59914 (19)	0.22777 (12)	0.0244 (3)	
H7	0.2892	0.5394	0.2131	0.029*	
C6	0.4733 (3)	0.67923 (19)	0.34378 (12)	0.0256 (4)	
C1	0.3324 (3)	0.6415 (2)	0.40945 (13)	0.0340 (4)	
C2	0.3630 (4)	0.7086 (3)	0.51432 (15)	0.0489 (5)	
H2	0.2637	0.6808	0.5549	0.059*	
C3	0.5406 (4)	0.8159 (3)	0.55754 (15)	0.0545 (6)	
H3	0.5623	0.8621	0.6280	0.065*	
C8	0.4401 (3)	0.7173 (2)	0.16603 (12)	0.0272 (4)	
C22	0.2927 (3)	0.8338 (2)	0.17803 (13)	0.0333 (4)	
C9	0.5883 (3)	0.72374 (19)	0.10034 (12)	0.0270 (4)	
C10	0.7341 (3)	0.49354 (19)	0.12257 (12)	0.0258 (4)	
C15	0.8841 (3)	0.3873 (2)	0.09093 (13)	0.0306 (4)	
H15	0.9778	0.4028	0.0446	0.037*	
C4	0.6873 (4)	0.8561 (3)	0.49742 (15)	0.0482 (5)	
H4	0.8094	0.9282	0.5269	0.058*	
C5	0.6524 (3)	0.7886 (2)	0.39261 (13)	0.0333 (4)	

### 2-Amino-3-cyano-4-(2,6-dichlorophenyl)-7-hydroxy-4H-\ benzo[1,2-b]pyran (II) Atomic displacement parameters (Å^2^)

**Table:**

	*U*^11^	*U*^22^	*U*^33^	*U*^12^	*U*^13^	*U*^23^
Cl1	0.0390 (3)	0.0566 (3)	0.0404 (3)	−0.0157 (2)	0.0042 (2)	0.0129 (2)
Cl2	0.0435 (3)	0.0712 (4)	0.0448 (3)	−0.0174 (2)	0.0084 (2)	0.0216 (3)
O1	0.0615 (10)	0.0440 (8)	0.0580 (9)	0.0319 (7)	0.0295 (7)	0.0303 (7)
O2	0.0421 (7)	0.0305 (6)	0.0376 (7)	0.0150 (5)	0.0189 (5)	0.0204 (5)
N1	0.0546 (11)	0.0603 (12)	0.0685 (12)	0.0324 (10)	0.0294 (9)	0.0405 (10)
N2	0.0469 (9)	0.0356 (8)	0.0409 (9)	0.0166 (7)	0.0178 (7)	0.0231 (7)
C14	0.0388 (10)	0.0269 (8)	0.0303 (9)	0.0110 (7)	0.0057 (7)	0.0088 (7)
C13	0.0433 (10)	0.0254 (8)	0.0340 (9)	0.0057 (7)	0.0052 (8)	0.0154 (7)
C12	0.0362 (9)	0.0298 (9)	0.0276 (8)	0.0013 (7)	0.0063 (7)	0.0128 (7)
C11	0.0289 (8)	0.0243 (8)	0.0222 (8)	0.0023 (6)	0.0012 (6)	0.0071 (6)
C7	0.0235 (8)	0.0264 (8)	0.0242 (8)	0.0026 (6)	0.0033 (6)	0.0090 (6)
C6	0.0285 (8)	0.0259 (8)	0.0247 (8)	0.0063 (7)	0.0043 (6)	0.0098 (7)
C1	0.0354 (10)	0.0376 (10)	0.0314 (9)	0.0017 (8)	0.0065 (7)	0.0139 (8)
C2	0.0648 (14)	0.0541 (12)	0.0293 (10)	−0.0009 (11)	0.0165 (9)	0.0143 (9)
C3	0.0846 (17)	0.0510 (13)	0.0232 (9)	−0.0059 (12)	0.0054 (10)	0.0076 (9)
C8	0.0309 (9)	0.0284 (8)	0.0249 (8)	0.0082 (7)	0.0036 (7)	0.0107 (7)
C22	0.0358 (9)	0.0374 (10)	0.0341 (9)	0.0099 (8)	0.0097 (7)	0.0191 (8)
C9	0.0325 (9)	0.0252 (8)	0.0255 (8)	0.0085 (7)	0.0032 (7)	0.0094 (7)
C10	0.0328 (9)	0.0232 (8)	0.0248 (8)	0.0055 (7)	0.0043 (7)	0.0113 (6)
C15	0.0362 (9)	0.0304 (9)	0.0299 (9)	0.0092 (7)	0.0109 (7)	0.0130 (7)
C4	0.0604 (13)	0.0444 (11)	0.0323 (10)	−0.0108 (10)	−0.0050 (9)	0.0073 (9)
C5	0.0375 (10)	0.0336 (9)	0.0298 (9)	0.0002 (7)	0.0057 (7)	0.0118 (7)

### 2-Amino-3-cyano-4-(2,6-dichlorophenyl)-7-hydroxy-4H-\ benzo[1,2-b]pyran (II) Geometric parameters (Å, º)

**Table:**

Cl1—C5	1.7383 (19)	C11—C7	1.515 (2)
Cl2—C1	1.736 (2)	C7—C8	1.515 (2)
O1—C14	1.362 (2)	C7—C6	1.538 (2)
O1—HO1	0.85 (3)	C7—H7	0.9800
O2—C9	1.353 (2)	C6—C5	1.394 (3)
O2—C10	1.392 (2)	C6—C1	1.397 (2)
N1—C22	1.144 (2)	C1—C2	1.383 (3)
N2—C9	1.342 (2)	C2—C3	1.364 (3)
N2—HNB	0.8600	C2—H2	0.9300
N2—HNA	0.8600	C3—C4	1.373 (3)
C14—C15	1.381 (2)	C3—H3	0.9300
C14—C13	1.390 (3)	C8—C9	1.354 (2)
C13—C12	1.380 (3)	C8—C22	1.413 (2)
C13—H13	0.9300	C10—C15	1.380 (2)
C12—C11	1.392 (2)	C15—H15	0.9300
C12—H12	0.9300	C4—C5	1.384 (3)
C11—C10	1.378 (2)	C4—H4	0.9300
			
C14—O1—HO1	112 (2)	C2—C1—Cl2	116.30 (15)
C9—O2—C10	118.64 (13)	C6—C1—Cl2	120.20 (14)
C9—N2—HNB	120.0	C3—C2—C1	119.25 (19)
C9—N2—HNA	120.0	C3—C2—H2	120.4
HNB—N2—HNA	120.0	C1—C2—H2	120.4
O1—C14—C15	116.39 (16)	C2—C3—C4	120.18 (19)
O1—C14—C13	123.88 (16)	C2—C3—H3	119.9
C15—C14—C13	119.73 (16)	C4—C3—H3	119.9
C12—C13—C14	119.48 (15)	C9—C8—C22	115.95 (15)
C12—C13—H13	120.3	C9—C8—C7	123.49 (14)
C14—C13—H13	120.3	C22—C8—C7	120.51 (15)
C13—C12—C11	122.22 (16)	N1—C22—C8	177.10 (19)
C13—C12—H12	118.9	N2—C9—O2	110.03 (14)
C11—C12—H12	118.9	N2—C9—C8	126.71 (15)
C10—C11—C12	116.26 (15)	O2—C9—C8	123.25 (15)
C10—C11—C7	122.30 (14)	C11—C10—C15	123.29 (15)
C12—C11—C7	121.44 (15)	C11—C10—O2	122.40 (14)
C11—C7—C8	109.10 (13)	C15—C10—O2	114.31 (14)
C11—C7—C6	111.28 (13)	C10—C15—C14	118.96 (16)
C8—C7—C6	114.32 (14)	C10—C15—H15	120.5
C11—C7—H7	107.3	C14—C15—H15	120.5
C8—C7—H7	107.3	C3—C4—C5	119.6 (2)
C6—C7—H7	107.3	C3—C4—H4	120.2
C5—C6—C1	114.50 (15)	C5—C4—H4	120.2
C5—C6—C7	124.05 (14)	C4—C5—C6	123.02 (17)
C1—C6—C7	121.41 (15)	C4—C5—Cl1	116.49 (15)
C2—C1—C6	123.48 (18)	C6—C5—Cl1	120.49 (13)
			
O1—C14—C13—C12	178.13 (17)	C10—O2—C9—N2	−176.56 (14)
C15—C14—C13—C12	−1.9 (3)	C10—O2—C9—C8	4.5 (2)
C14—C13—C12—C11	−0.1 (3)	C22—C8—C9—N2	1.8 (3)
C13—C12—C11—C10	2.0 (2)	C7—C8—C9—N2	−175.57 (16)
C13—C12—C11—C7	−177.63 (15)	C22—C8—C9—O2	−179.42 (15)
C10—C11—C7—C8	8.4 (2)	C7—C8—C9—O2	3.2 (3)
C12—C11—C7—C8	−172.08 (15)	C12—C11—C10—C15	−2.0 (2)
C10—C11—C7—C6	−118.71 (17)	C7—C11—C10—C15	177.61 (15)
C12—C11—C7—C6	60.9 (2)	C12—C11—C10—O2	178.39 (15)
C11—C7—C6—C5	70.1 (2)	C7—C11—C10—O2	−2.0 (2)
C8—C7—C6—C5	−54.1 (2)	C9—O2—C10—C11	−5.0 (2)
C11—C7—C6—C1	−107.41 (18)	C9—O2—C10—C15	175.30 (15)
C8—C7—C6—C1	128.44 (17)	C11—C10—C15—C14	0.1 (3)
C5—C6—C1—C2	1.3 (3)	O2—C10—C15—C14	179.77 (15)
C7—C6—C1—C2	179.01 (18)	O1—C14—C15—C10	−178.14 (16)
C5—C6—C1—Cl2	−177.27 (13)	C13—C14—C15—C10	1.9 (3)
C7—C6—C1—Cl2	0.4 (2)	C2—C3—C4—C5	0.9 (4)
C6—C1—C2—C3	−0.9 (3)	C3—C4—C5—C6	−0.3 (3)
Cl2—C1—C2—C3	177.78 (18)	C3—C4—C5—Cl1	−179.69 (18)
C1—C2—C3—C4	−0.3 (4)	C1—C6—C5—C4	−0.7 (3)
C11—C7—C8—C9	−9.1 (2)	C7—C6—C5—C4	−178.35 (17)
C6—C7—C8—C9	116.24 (18)	C1—C6—C5—Cl1	178.63 (13)
C11—C7—C8—C22	173.69 (15)	C7—C6—C5—Cl1	1.0 (2)
C6—C7—C8—C22	−61.0 (2)		

### 2-Amino-3-cyano-4-(2,6-dichlorophenyl)-7-hydroxy-4H-\ benzo[1,2-b]pyran (II) Hydrogen-bond geometry (Å, º)

**Table:**

*D*—H···*A*	*D*—H	H···*A*	*D*···*A*	*D*—H···*A*
C7—H7···Cl2	0.98	2.50	3.078 (3)	117
N2—H*NB*···O1^i^	0.86	2.19	3.048 (2)	173
O1—H*O*1···N1^ii^	0.85 (3)	1.95 (3)	2.762 (2)	160 (3)

Symmetry codes: (i) −*x*+2, −*y*+1, −*z*; (ii) *x*+1, *y*−1, *z*.
